# P-1151. Clinical Impact of Susceptibility Testing in Anaerobic Infections from Pediatric Patients

**DOI:** 10.1093/ofid/ofae631.1337

**Published:** 2025-01-29

**Authors:** Diego A Cruz Vidal, Raimey Benson, Huanyu Wang, Sophonie J Oyeniran

**Affiliations:** Nationwide Children's Hospital, Columbus, Ohio; Nationwide Children's Hospital, Columbus, Ohio; Nationwide Children's Hospital, Columbus, Ohio; Nationwide Children's Hospital, Columbus, Ohio

## Abstract

**Background:**

Anaerobic infections are rarely described in pediatric populations, where routine use of anaerobic blood cultures remains controversial. Due to rising resistance, susceptibility to some anti-anaerobic drugs is unpredictable, yet anaerobic antimicrobial susceptibility testing (AST) is not widely available.

Herein we characterize anaerobic isolates recovered from subjects treated at Nationwide Children's Hospital (NCH) and describe the impact of anaerobic culture and AST on prescribing practices.

**Figure:**
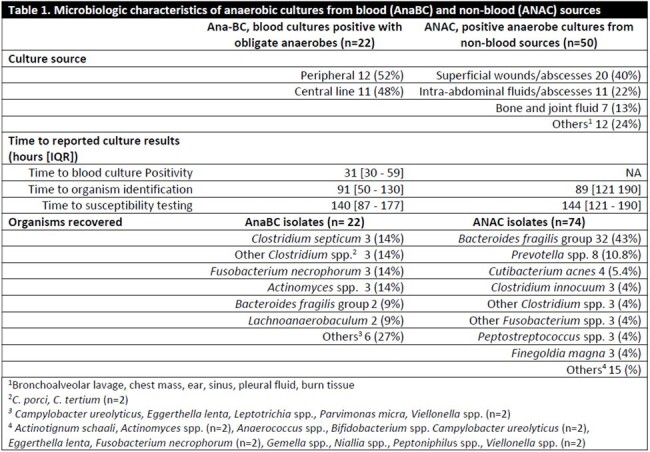

**Methods:**

All anaerobic cultures from non-blood sources (ANAC) and blood cultures with obligate anaerobic organisms (AnaBC) during 2023 were identified. At NCH, anaerobic bottles are collected for all BC. Anaerobic isolates are identified by molecular methods and AST performed on sterile sites or by request. Prescribing practices for subjects with anaerobic isolates and AST performed were reviewed.

**Figure:**
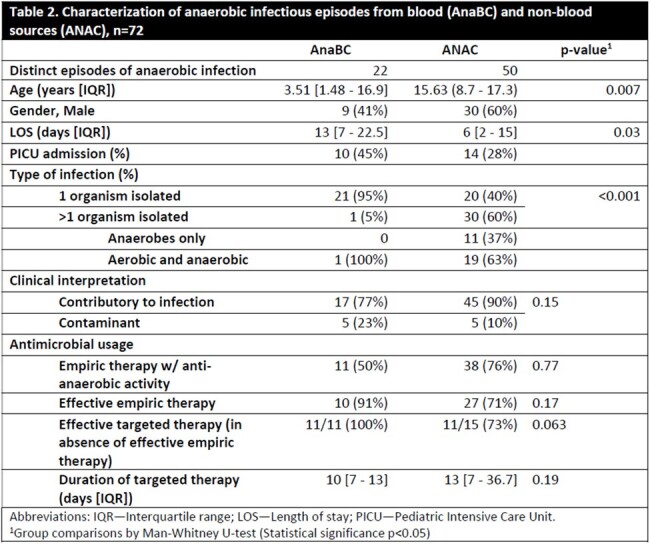

**Results:**

In 2023, AST was performed on 74 ANAC isolates (25% positive cultures) and 22 AnaBC isolates (70%) from 72 distinct infectious episodes. ANAC isolates were mostly from superficial wounds/abscesses (40%) and intra-abdominal sources (22%), while most AnaBC isolates were from peripheral cultures (52%). *Bacteroides fragilis* group (43%) and *Clostridium* spp. (28%) were the most frequent organisms in ANAC and AnaBC, respectively. Time to identification (89 h) and time to AST (143 h) did not differ between groups (Table 1).

Of the 72 infectious episodes, 38 (54%) were from males with a median age of 14.7 years; 22 (31%) were admitted to the intensive care unit (ICU) and 7 (10%) had immunocompromise at baseline. In comparing infectious episodes from AnaBC or ANAC, episodes from AnaBC had significantly younger subjects (3.5 vs. 15.6 years), longer lengths of stay (13 vs. 6 days) and were more commonly mono-microbial (95% vs. 40%). While not significant, episodes from AnaBC tended to have higher rates of effective empiric (91% vs. 71%) and targeted (100% vs. 73%) therapy than those from ANAC (Table 2).

**Conclusion:**

Gaps in antimicrobial optimization were observed for ANAC infectious episodes. Low rates of effective empiric therapy in these settings indicates the need for timely anaerobic AST. Rapid availability of results may improve rates of effectively treated anaerobic infections.

**Disclosures:**

**All Authors**: No reported disclosures

